# Fulminant type 1 diabetes mellitus in pregnancy

**DOI:** 10.1590/1414-431X20209633

**Published:** 2020-07-17

**Authors:** Chun-Yu Li, Yang Li, Zi-Ying You, You-Ren Liu, Yu-Jue Wang, Tong Mu, Cheng-Zhi Zhao, Zu-Mu Yi, Yu-Wei Zhang

**Affiliations:** 1 West China School of Medicine, Sichuan University, Chengdu, China; 2 Department of Endocrinology and Metabolism, West China Hospital, Sichuan University, Chengdu, China; 3 Sichuan Provincial Academy of Medical Sciences & Sichuan Provincial People's Hospital Health Management Center, Chengdu, China; 4 Department of Obstetrics and Gynecology, Sichuan Provincial Academy of Medical Sciences & Sichuan Provincial People's Hospital, Chengdu, China

**Keywords:** Fulminant type 1 diabetes mellitus, Pregnancy, Diabetic ketoacidosis, Plasma glucose, Hemoglobin A1C

## Abstract

Fulminant type 1 diabetes mellitus (FT1DM) has received clinical attention for its low incidence and poor prognosis. Currently, few cases of FT1DM are associated with pregnancy in clinical practice, but it poses a great threat to the life of mothers and infants. Here, we present two cases of FT1DM in pregnancy. In Case 1, the patient was a 26-year-old woman who was admitted to the hospital with reduced fetal movement. She was diagnosed with FT1DM and delivered a dead female fetus. Lispro and lantus were administered to control blood glucose, and lipoic acid for antioxidant therapy. In Case 2, the patient was a 28-year-old woman who developed nausea, vomiting, diarrhea, and polydipsia, which later proved to be FT1DM. An abortion was induced and blood glucose levels were controlled using an insulin pump. All physicians should be aware of this disease in order to provide prompt diagnosis and emergency treatment, thus improving maternal prognosis. We suggest that plasma glucose/hemoglobin A1C ratio be adopted as a new clinical parameter in predicting FT1DM.

## Introduction

Type 1 diabetes mellitus (T1DM) can be divided into two categories: the autoimmune type (type 1A) and the spontaneous type (type 1B). The American Diabetes Association and the World Health Organization classify fulminant type 1 diabetes mellitus (FT1DM) as a subtype of type 1B. FT1DM was first described in Japan and since then has been reported in other parts of East and South-East Asia. FT1DM was first reported by Imagawa et al. (1), and was characterized by an extremely rapid progression of hyperglycemia and diabetic ketoacidosis (DKA) due to the almost complete destruction of pancreatic beta-cells. The diagnostic criteria for FT1DM were reported by the Committee of the Japan Diabetes Society in 2012 (2). FT1DM is confirmed when: 1) occurrence of DKA or ketoacidosis soon after (approximately 7 days) the onset of hyperglycemic symptoms (elevation of urinary and/or serum ketone bodies at first visit); 2) plasma glucose (PG) level ≥16.0 mM and hemoglobin A1C (HbA1C) level <8.7% at first visit; and 3) urinary C‐peptide excretion <10 μg/day or fasting serum C‐peptide level <0.3 ng/mL (0.10 nM) and <0.5 ng/mL (0.17 nM) after intravenous glucagon (or after meal) load at onset. According to Imagawa et al. (3), FT1DM accounts for about 20% of ketosis-onset T1DM in Japan. FT1DM can occur during pregnancy and immediately after delivery (2). In a national study of Japan, 21% of FT1DM occurred in pregnant women, 14 times the rate of typical T1DM (3).

We report two cases of FT1DM during pregnancy to investigate the etiology, diagnosis, treatment, and the maternal and fetal prognosis of the disease, as well as the clinical application of PG/HbA1C ratio. The two cases were at different gestational weeks and both had symptoms of infection and ended with severe consequences such as stillbirth. Moreover, Case 2 was delayed in another hospital for about a week before accurate diagnosis and corresponding treatment. The question raised is whether the current focus on FT1DM is enough.

### Case report 1

The patient was a 26-year-old women, G1P0 (G: gestation, P: parturition), 38 weeks of gestation without a history of pregnancy or family history of diabetes. On May 8, 2017, she was admitted to the hospital due to 38 weeks of gestation and over 10 h of decreased fetal movement. A color Doppler ultrasonography (Soering, Germany) showed no fetal heartbeat, suggesting intrauterine stillbirth. Laboratory results showed that PG was 29.43 mM, pH was 7.172, urine glucose was 4+, urine ketone was 3+. Other parameters are shown in Table 1. She was diagnosed with DKA and intrauterine stillbirth. After admission, she was given oxygen, intravenous insulin, sodium bicarbonate, and a large amount of fluid replacement therapy.

**Table 1:** Laboratory data of Patient 1.

Laboratory data on May 8		Laboratory data on May 18	
White blood cells	11.92×109/L	White blood cells	8.59×109/L
Red blood cells	4.10×1012/L	Red blood cells	3.23×1012/L
Hemoglobin	118.00 g/L	Hemoglobin	93 g/L
Platelets	247×109/L	Platelets	402×109/L
Neutrophils	80.30%	Hematocrit	0.295
hCRP	87.00 mg/L	Ca	2.16 mM
Na	128.0 mM	AST/ALT	0.9
Cl	85.0 mM	Fasting C-peptide	<0.01 ng/mL
Fe	44.9 µM	2-h C-peptide	<0.01 ng/mL
ALT	40.7 U/L	Amylase	114 U/L
LDH	468.0 U/L	Lipase	225.0 U/L
GGT	55.0 g/L	ALP	139 U/L
ALP	159.0 U/L	Creatinine	39.8 µM
Uric acid	425.0 µM	Glucose	6.59 mM
Glucose	29.43 mM	Total protein	58.0 g/L
Lactic acid	2.3 mM	Albumin	31.8 g/L
pH	7.172	Prothrombin time	11.0 s
pO_2_	104.2 mMHg	D-Dimer	1.93 mg/L
pCO_2_	14 mMHg	FDP	5.6 Ug/mL
Base excess	-21 mM	Urine pH	6
Fibrinogen	7.10 g/L	Urine Blood	3+
Prothrombin time	11.00 s	Urine white blood cells	2+
Urine pH	5.5	Urine amylase	575 U/L
Urine glucose	4+	Urine glucose	-
Urine ketone	3+	Urine ketone	-

hCRP: hypersensitive C-reactive protein; AST: aspartate aminotransferase; ALT: alanine aminotransferase; LDH: lactate dehydrogenase; GGT: γ-glutamyl transpeptidase; ALP: alkaline phosphatase; pH: potential of hydrogen; pO_2_: partial pressure of oxygen; pCO_2_: partial pressure of carbon dioxide; FDP: fibrin degradation products.

At 6 pm on May 8, she was transferred to obstetrics. Her random blood glucose was 21.9 mM, urine ketone was 4+, and HbA1C was 5.4%. After admission, she was treated in the ICU, insulin was pumped to control blood glucose, and sulbenicillin (FUAN Pharmaceutical, China) was used for anti-infection therapy. On May 10, rivanol (HEFENG Pharmaceutical, China) was injected into the amniotic cavity to induce an abortion. During the period, blood glucose fluctuated greatly (fasting blood glucose 4.9–22.6 mM), which was adjusted repeatedly. On May 17, the blood glucose variability was between 4.0 and 12 mM, blood amylase was 102U/L, lipase was 183U/L, urine occult blood was 3+, urine ketone was 2+, and urine glucose was 3+.

On May 18, she was admitted to the endocrinology department with a fingertip blood glucose of 6.0 mM, and blood ketone of 0.2 mM. The results of laboratory examination are reported in Table 1. She was diagnosed as FT1DM, and was given lispro (Eli Lilly and Company, USA) and lantus (Aventis Pharma Deutschland GmbH, Germany) to control blood glucose levels and lipoic acid (Hameln Pharmaceuticals GmbH, Germany) for antioxidant therapy. She recovered after 20 days of therapy and was discharged.

### Case report 2

The patient was a 28-year-old Chinese woman with unremarkable family history. She was diagnosed with gestational diabetes during her first pregnancy with a fasting blood glucose of 5.2 mM and received diet therapy. Later, she gave birth to a healthy full-term baby and the tests following the gestational period revealed normal glucose. This was her second pregnancy and at 24 weeks' gestation, she complained of nausea, vomiting, and diarrhea for about 1 week, however, she did not seek any medical help. Two days later, she developed severe polydipsia and went to a general hospital but no diagnosis was made. She was then transferred to our hospital.

On admission, her PG was as high as 31.33 mM, serum pH was 7.273, urine ketone was 4+, urine glucose was 4+. Other parameters are reported in Table 2. Arterial blood gas analysis demonstrated severe metabolic acidosis with normal electrolytes. A diagnosis of DKA was made and treatment with saline and intravenous insulin was initiated immediately. However, she developed prelabor rupture of membranes and an abortion was induced. Additional tests showed negative antiglutamic acid decarboxylase antibody, insulin islet antibody, and insulin autoantibody. Fasting C-peptide was 0.003 nM and HbA1c was 5.4%. Serum lipase was slightly elevated (66 IU/L) and serum amylase was otherwise normal. The patient was diagnosed with FT1DM. She was administered insulin by a pump and blood glucose was under good control. On March 3, her serum glucose was 5.2 mM, pH was 7.439, urine glucose and urine ketone were all negative, and the other parameters are shown in Table 2. She was discharged after 30 days of treatment.

**Table 2:** Laboratory data of Patient 2.

Laboratory data on May 8		Laboratory data on March 3	
White blood cells	37.61×10^9^/L	White blood cells	5.56×10^9^/L
Red blood cells	4.86×10^12^/L	Red blood cells	3.99×10^12^/L
Hemoglobin	153.0g/L	Hemoglobin	123 g/L
Platelets	187×10^9^/L	Platelets	304×10^9^/L
Neutrophils	80.0%	Neutrophils	59.1%
hCRP	5.81 mg/L	hCRP	2.0 mg/L
Na	132.9 mM	Na	144.8 mM
Cl	100.4 mM	HbA1c	5.4%
K	5.13 mM	K	4.11 mM
Triglycerides	4.99 mM	Triglycerides	1.02 mM
ALP	101 U/L	Lipase	66 IU/L
Uric acid	741.0 µM	Fasting C-peptide	0.004 ng/mL
Glucose	31.33 mM	Glucose	5.2 mM
Lactic acid	2.6 mM	Lactic acid	1.2 mM
pH	7.273	pH	7.439
pO_2_	110 mmHg	pO_2_	82.8 mmHg
pCO_2_	19.7 mmHg	pCO_2_	39.2 mmHg
Base excess	-16.1 mM	Base excess	1.8 mM
Anion gap	8.1 mM	Glycated albumin	19.17%
Prothrombin time	14.8 s	GADA	Negative
D-Dimer	19.08 mg/L	IAA	Negative
INR	1.26	ICA	Negative
Urine pH	5.5	Urine pH	5.5
Urine glucose	4+	Urine glucose	-
Urine ketone	4+	Urine ketone	-

CRP: C-reactive protein; HbA1c: Hemoglobin; A1cALP: alkaline phosphatase; pH: potential of hydrogen; pO_2_: partial pressure of oxygen; pCO_2_: partial pressure of carbon dioxide; GADA: glutamic acid decarboxylase antibody; IAA: insulin autoantibody; INR: international normalized ratio; ICA: islet cell autoantibody.

## Discussion

More than 90% of FT1DM patients are adults, and the incidence of FT1DM is similar between males and females, with Japanese individuals having the highest incidence (3). At present, the pathogenesis of FT1DM has not been clarified, which may be related to hereditary susceptibility (4), viral infection (5), autoimmunity, pregnancy, and drug-induced hypersensitivity syndrome. It is reported that most patients who develop T1DM in pregnancy have FT1DM (6), which may be caused by significant changes in the immunological milieu during pregnancy (7), and often leads to fetal abortion and stillbirth (8). Shimizu et al. (9) compared the clinical characteristics of pregnancy-associated fulminant type 1 diabetes (PF) and non-pregnant fulminant type 1 diabetes (NPF), and found that severe acidosis and increased infection occurred in the former owing to abnormal hormone levels and metabolism. Although the specific mechanisms of FT1DM in relation to pregnancy have not been clarified, viral infection and human leukocyte antigen are considered to be involved. In Case 1, the patient was infected with Epstein-Barr virus preceding disease onset, while in Case 2, the patient was suspected of having acute gastroenteritis, which may be related to the development of FT1DM.

In both cases, the patients delivered dead fetuses and were discharged after several days of therapy. Shimizu et al. (9) reported that fetal death occurred in 12 of 18 PF patients (67%). Among the 6 live-born infants, 83.3% had flu-like symptoms and 50% had abdominal symptoms. Meanwhile, the mean duration of the hyperglycemic symptoms was 1.8 days (1–4), the mean PG level was 608 mg/dL (372-867), mean HbA1c was 5.6% (5.3-5.9%), and mean amylase was 3.8 (0.7–7.7), multiples of the upper limit of the normal range.

In Case 1, the onset of the disease was acute, and the time from clinical manifestations to therapy was less than 1 day. Admission tests revealed stillbirth and DKA. Her serum lipase and serum amylase levels were high. Her initial blood glucose was 29.43 mM, HbA1C was 5.4% on May 8, fasting C-peptide <0.01 ng/mL, and postprandial 2-h C-peptide <0.01 ng/mL, which were consistent with the diagnosis of FT1DM. In Case 2, the suspected acute gastroenteritis may have been the trigger and the disease progressed acutely in 2 days, resulting in death of the infant. She had elevated liver function and serum lipase test results. Her diagnosis of FT1DM was confirmed with an admission blood glucose of 30 mM, HbA1c of 5.4%, and fasting C-peptide of 0.003 nM. Delay in diagnosis and treatment can bring tremendous mental and physical trauma to a patient. Therefore, it is crucial for physicians to test blood glucose of a pregnant woman with even sub-clinical or minimally symptomatic infection when abnormal fetal movements are present.

Aleppo et al. (10) showed that for T1DM patients, about 63% of the 24-h glucose levels were in the target glucose range (3.9–10.0 mM). Analogously, Ying L et al. (11) assessed glucose levels of FT1DM through continuous glucose monitoring. They found that only 49.8% at 24 h were in the target glucose range of 3.9–10.0 mM, suggesting a relatively worse glucose control than T1DM. They also reported the dynamic glucose features of FT1DM with the patients presenting higher glucose levels and a large glucose fluctuation. Besides, pregnancy status appeared to lower the average glucose levels without affecting glucose fluctuation. These can facilitate the clinical diagnosis and therapy of FT1DM.

HbA1C is considered the gold standard for evaluation of the blood glucose control level and condition of diabetes mellitus. However, the level of HbA1C does not increase during the acute onset of FT1DM, making it inadequate for the evaluation of FT1DM. Liu et al. (12) reported that HbA1C was nearly normal and PG was significantly increased in PF compared with DKA patients. This study also found that a PG/HbA1C ratio with a threshold of ≥3.3 could be used as a cut-off point for predicting PF from DKA, providing 89% sensitivity and 100% specificity. Another study (13) indicated that a PG/HbA1C ratio with a threshold of ≥4.2 was useful to identify DKA patients at high risk of FT1DM, with 94% sensitivity and 98% specificity. These data have profound implications for the prevention of FTIDM. In Case 1, the PG/HbA1C ratio of the patient was 5.45, in Case 2, it was 5.56, confirming the above data. Whether this ratio can be applied to the clinic remains to be further studied. As for the treatment of FT1DM, continuous subcutaneous injection of insulin is the first choice, since the patient's islet beta cells are almost completely destroyed and blood glucose levels fluctuate greatly, making it difficult to control (14).

In conclusion, the PG/HbA1C ratio is of great significance and deserves more studies as a new clinical parameter for predicting FT1DM. Although the mechanism of FT1DM remains unclear and its incidence remains relatively low in China, it has acute onset, rapid progression, and poor prognosis, and should be promptly diagnosed to receive treatment. Thus, we specifically call for clinical attention for FT1DM.
